# Metaphor, multimodality and generative AI: Synthetic images prompted by figurative expressions in anti-immigration discourse

**DOI:** 10.1371/journal.pone.0359980

**Published:** 2026-10-16

**Authors:** Christopher Hart

**Affiliations:** Department of Linguistics and English Language, Lancaster University, Lancaster, Lanacshire, United Kingdom of Great Britain and Northern Ireland; Universitas Prima Indonesia, INDONESIA

## Abstract

Complex social processes like migration are frequently understood via metaphors. In right-wing media and far-right political discourses, two metaphors that abound draw on flood and invasion frames to problematise immigration and legitimise hostile attitudes and actions towards it. In so far as metaphor involves the construction of mental imagery, it is intimately bound with visual experience, including semiotically mediated visual experience. In a semiotic landscape increasingly populated by synthetic images, I argue that the meaning of metaphors may be modulated by AI-generated visual content and that this is especially important in contexts of political communication. The study investigates images generated by prompts instantiating an immigration is flood versus an immigration is invasion metaphor to inquire if and how they differ by source-frame and what are the ideological implications of the images returned. Following an initial pilot study, several specific semiotic features are analysed, including colour, properties of the figure (size, shape, orientation) and ground elements (environment, securitising features and frame-consistent features like fire and water). Results show significant differences between metaphorical frames and highlight the potential that AI-generated images have to reflect and reinforce ideological positions.

## 1. Introduction

Complex social processes like migration are frequently understood via metaphors [1]. In right-wing media and far-right political discourses, two metaphors that abound draw on flood and invasion frames to problematise immigration and legitimise hostile attitudes and actions towards it [2–9]. In so far as metaphor involves the construction of mental imagery [10–13], it is intimately bound with visual experience, including semiotically mediated visual experience. In a semiotic landscape increasingly populated by synthetic images, I argue that the meaning of metaphors may be modulated by AI-generated visual content and that this is especially important in contexts of political communication. The study investigates images generated by prompts instantiating an immigration is flood versus an immigration is invasion metaphor to show how they differ by source-frame and what the implications are of the images returned.

## 2. Background

Generative AI (Gen-AI) has had a transformative effect on the way people engage with the world around them, including how they interact with objects, other people, language and ideas. Gen-AI is incorporated into workflows across a variety of industries. In areas like advertising, the use of Gen-AI has fundamentally changed the semiotic landscape [14,15]. The use of AI-generated images in news production has also significantly increased [16]. However, the accessibility of Gen-AI technology means it can also be used recreationally by almost anyone for purposes of discovery or creativity. In the UK, 73% of the public report using Gen-AI in their day-to-day life (https://www.gov.uk/government/publications/ai-skills-for-life-and-work-general-public-survey-findings/ai-skills-for-life-and-work-general-public-survey-findings). This, too, has shifted the semiotic landscape as users share AI-produced content via digital social media platforms. Some of the most staggering statistics pertain to visual content. It is estimated that, since the launch of DALL·E 2 in 2022, over 15 billion images have been produced by text-to-image (TTI) generators with 34 million images currently being created every day (https://journal.everypixel.com/ai-image-statistics). As a result of this activity, 71% of images found on social media are now AI-generated (https://journal.everypixel.com/ai-image-statistics). The consequence of this is that the visual representation of salient social and political events like immigration and war is increasingly mediated by Gen-AI and the human-archival-mechanistic entanglement it has created [17].

The industrial production of AI-generated content as well as regular user engagement with Gen-AI technology has led to both creative opportunities and societal challenges [18,19]. Major challenges include fake news [20–22] and the reproduction of social biases [23–27]. For example, Bianchi et al. [23] found that, across a broad range of prompts, AI generates images that reinforce racial and gendered stereotypes, promote whiteness as an ideal and reproduce American norms, while prompts mentioning specific social groups (e.g., by race or nationality) produce images “that tie specific groups to negative or taboo associations like malnourishment, poverty, and subordination” (p. 1495). Hart [24] showed that AI images of immigrants and refugees in motion tend to depict the subject as a large, unbounded mass moving toward the viewer through a natural or agricultural environment. This stereotypical image reflects and reinforces the discursive construction of migration as a substantive and unrelenting ‘problem’ and ignores both the circumstances that motivate migration and the positive outcomes of migration.

AI text-to-image (TTI) generation works as a user feeds a latent diffusion model a textual prompt. For lay users, this will often be via a chatbot like ChatGPT (https://chatgpt.com), Google Gemini (https://gemini.google.com) or Claude (https://claude.ai) or a third-party platform or application like Canva (https://www.canva.com). Original models launched in 2022 include Stable Diffusion, Midjourney and DALL·E (which previously powered ChatGPT’s TTI capabilities). However, since then many more models have been developed, including GPT-Image-1.5 (which succeeds DALL·E 3 to provide current TTI capabilities for ChatGPT) and Nano Banana Pro (which enables TTI generation in Google Gemini). Models are trained on humanly produced data from the internet (and increasingly AI-produced data on the internet). A TTI model uses the data made available to it (billions of images and associated text on the internet) to learn relationships between text and images. It then uses those patterns to predict what the scene described by a given word or phrase looks like so that when then given a specific prompt it is able to generate an image to fit. The result, as Cope and Kalantzis ([28]: 124) state, “is a uniquely reconstituted digital artifact – in text, image, sound or multimodal combination”. The model itself does not recognise meaning and works only by attaching statistical weight to connections established within a vector space. This, however, does not mean that the patterns it detects are not meaningful for humans or that the newly created image does not itself become a meaningful artefact in the world.

Westberg and Kvåle ([29]: 2) describe AI text-to-image generation as a “semiotic technology”, that is, “a technology for meaning making that is deeply inscribed with certain sets of social norms, values and ideologies”. AI-generated images will naturally reflect the social identities, values and stereotypes enshrined in the images on which models are trained. Exposure to AI-generated images may therefore serve to reconfirm existing ideas, ideologies and prejudices. And ultimately, since AI image-generation leads to a homogeneity of images [30], alternative perspectives may be squeezed out. As Jungherr and Schroeder ([31]: 5) state, AI-based systems cause discourse, over time, to “include fewer central topics, voices, and positions, with non-conforming content pushed out of sight”.

Laba et al. [17] address the role of AI-generated images in curating conflict and show how images produced through AI to represent the Russian invasion of Ukraine “render a fabricated vision of historical events which increasingly becomes part of collective memory” (p.3). Crucially, the AI-generated images analysed tended to offer a sanitised view that excludes critical aspects such as death, injury and the suffering of children and refugees. The images also focussed disproportionately on urban areas thus failing to capture the full scope of the conflict. Laba et al. [17] argue that AI-generated images introduce a new genre that blends documentation with speculative fiction. Pawelec [32] argues that synthetic audio-visual content threatens core democratic functions and norms by creating the epistemic conditions that allow liars to prosper, that prevent proper deliberation, and that lead to the further marginalization of disempowered communities. The proliferation of synthetic images, moreover, leads to a distrust of authentic media so that seeing is no longer believing [32,33]. AI-generated images therefore constitute “important, but currently understudied, social texts to attend to in relation to complex social phenomena” ([26]: 2).

An all-important question concerns the data on which models are trained and its representativeness. However, training data is highly selective and skewed toward data originating in Europe and America [34,35]. AI-produced images therefore reflect the ideological perspectives and aesthetic preferences of the Global North and potentially present a version of the world seen through a Western or colonial gaze [36].

All of this makes the outputs of Gen-AI as well as the processes of production and consumption surrounding those outputs highly relevant for the field of Critical Discourse Studies (CDS) [37]. As Gillings et al. ([37]: 626) state, we now have a new type of semiotic data, produced by machines rather than humans, and this needs to be assessed critically in terms of its production, reception and social impact.

In the domain of politics, Gen-AI TTI technology is actively employed in the propagandistic promotion of ideologies [38,39]. For example, Lulu [38] analysed the AI-generated video *Trump Gaza,* which was shared on social media by Donald Trump. Visual themes expressed within the video include Gaza as a site of devastation and destruction caused by the war which is contrasted with a utopian vision of Gaza under Trump’s ownership as a paradise destination and place of luxury. AI-generated images depicting war from a particular perspective are shown to increase support for the actors whose perspective is presented [40]. This effect is reversed if participants are subsequently informed about the synthetic nature of the images (ibid.). However, in the case of journalistic images, the average person struggles to identify AI-generated content without disclaimers [41] and AI-generated visuals that are manifestly synthetic are judged as more reflective of a news article’s content and more strongly facilitate comprehension [42].

In the context of immigration, Gen-AI has been used by far-right groups to create images of immigrants which construct them as a cultural or corporeal threat (Hart & Voice forthcoming). This visual representation is consistent with the kind of verbal representation observed in right-wing and far-right media and political discourse, where migrants are described in terms of excessive quantities or as an alien or aggressive Other [43,44]. One specific linguistic feature implicated here is metaphor with migration frequently being described in terms of *floods* or *invasions* [2–5,8,9,45–47].

In cognitive linguistics, metaphor is treated as a cognitive process of frame-projection in which selected aspects of a source-frame are mapped to provide a template for thinking, feeling and acting with respect to a target-frame [48]. This produces *entailments* as relations obtaining between source-frame elements come, as a consequence of the mapping, to be held between equivalent elements in the target-frame. Psycholinguistic evidence suggests that metaphor is not an amodal process but a form of *simulation* that involves the construction of mental imagery (visual, auditory, kinetic) derived from source and target frames (see [11] for an overview). This has implications for the role of visual and other forms of sensory and motor experience in shaping what metaphors mean.

In CDS, metaphor is identified as a key cognitive process involved in the discursive reproduction of ideology and the attendant legitimation of social action [49–51]. Metaphors are shown to play an important structuring role across a range of discourses, including discourses of migration, where they influence perceptions, emotions, decisions and actions [52–55]. A source-frame that has persisted in anti-immigration discourse over time is the flood frame while the invasion frame represents a more recent innovation [9]. Both frames feature in the discourses of far-right actors and organisations as well as the right-wing press, as exemplified below:

(1) Over 100 migrants illegally entered the UK by small boat today. The invasion is on. (Nigel Farage, 16 May 2020, via Twitter)
(2) (200 British anarchists ‘help migrants storm 10 miles into Channel Tunnel’ during massive invasion from Calais. (dailymail.co.uk, 4 Nov. 2015)
(3) I warned about illegal migration when it was a trickle – years of failure have turned it into a flood (Nigel Farage, 27 May 2024, via telegraph.co.uk)
(4) Illegal migrants flooding into EU: Record 1.8MILLION border breaches in just one year. (express.co.uk/6 Apr. 2016)

In the UK, the invasion metaphor recently entered mainstream political discourse when it was used in parliament in 2022 by the then Home Secretary. The same speaker extended the flood frame to another form of natural disaster when she likened immigration to a hurricane. Such uses and extensions represent a radicalization of immigration rhetoric and/or a mainstreaming of far-right political discourse [56,57].

(5) Let’s be clear about what is really going on here: the British people deserve to know which party is serious about stopping the invasion on our Southern coast and which party is not. (Suella Braverman MP, 31 Oct. 2022)
(6) The wind of change that carried my own parents across the globe in the 20th century was a mere gust compared [with] the hurricane that is coming. (Suella Braverman MP, 3 Oct. 2023)

Metaphor is at root a cognitive process that is manifested in and activated by metaphorical expressions in discourse. As such, the range of expressive forms that are capable of evoking a metaphor extends to semiotic modes besides language, including images as well as gestures [49,58–62]. The figurative framing of immigration as a flood, for example, is rendered by the image of a massive funnel inscribed with the word ‘refugees’ [63] while arrows used to represent migration (in a way that recalls military maps) may realise an invasion framing [49]. Visually expressed metaphors are also shown to achieve framing effects [64,65].

It follows that in a multimodal text, language and image may enter into different intersemiotic relations with respect to metaphor [6,66]. A metaphor may be simultaneously expressed in language and image where source and target frames and the mappings between them are fully discernable in each mode (*cross-modal metaphor*). Alternatively, source and target frames may be expressed exclusively in different modes with any metaphorical reading dependent on an interaction between the two forms of representation (*intermodal metaphor*). Another possibility is that a metaphor is not explicitly rendered in the image but the image is itself capable of being read metaphorically in the context of a verbally expressed metaphor (in which case we may speak of *metaphor anchorage*). This potential often exists as a function of intertextual or interdiscursive links an image makes with the source-frame. A still further possibility is that, although the image may not be interpreted metaphorically, it nevertheless contains visual elements associated with the source-frame in a verbally expressed metaphor (in which case we may speak of *frame-consistent images*). In images, as El Refaie ([63]: 76) states, “the boundaries between the literal and the metaphorical are fuzzy and highly context-dependent”. Whether an image expresses a metaphor fully, partially, potentially or not at all is therefore dependent on the reader’s own subjectivity and is not inherent within the image itself. This leaves open the possibility that the same image will be understood literally by one individual and metaphorically by another.

Within the intersecting contexts of politics, multimodality and Gen-AI, what TTI models do with metaphorical prompts is interesting for several reasons. Previous research has addressed the metaphors used to talk about Gen-AI and its role in society [67] but not how Gen-AI handles metaphorical prompts and what this might mean for metaphor and society.

Exactly how TTI generation operates under the hood is, for most people working outside of computer science, something of a black box. This is especially the case with respect to metaphor. The elementary unit of analysis is the token. Typically, this is a word. However, it may be a smaller unit in the case of words with composite meanings or a larger phrase-level unit depending on how strongly the expression is represented in the training data. Whether a metaphorical expression is parsed compositionally or not therefore depends on both the metaphor and the model. If a metaphorical expression is parsed non-compositionally as a single unit, then visual outputs are more likely to reflect literal, authentic images of the kind that regularly co-occur with the figurative expression. Of course, images may still capture aspects of meaning encoded in a metaphor, such as images of large groups of people reflecting the sense of excessive quantity expressed by flood and invasion metaphors. However, if a metaphorical expression is parsed compositionally, then visual outputs are likely to be more creative, combining aspects of both source and target frames in a novel type of image. The purpose of this paper is to investigate the images that TTI models produce when given metaphorical prompts for immigration, including if and how the images vary by source-frame.

From a social perspective, the images returned may be reflective of what these metaphors mean for particular communities. AI-generated images represent a statistical composite of actual images that regularly co-occur with a given linguistic form. They may thus instantiate the visual component of a multimodal construction [68,69] which is then evoked by metaphorical expressions even in the absence of co-text images [24]. However, the images returned may equally be *effective* of meaning. As community members encounter AI-generated images in the context of metaphorical expressions, either by engaging with TTI technology themselves and providing metaphorical prompts or as they are exposed to multimodal texts produced by others which contain both metaphorical expressions and synthetic images prompted by those expressions, AI images may increasingly come to shape metaphorical meaning. That is, AI images may increasingly come to influence the kind of mental imagery that is activated by metaphorical expressions, reinforcing connections between source and target frames and confirming implications that arise as a result of the mapping. It is even conceivable that a metaphor considered ‘dead’ or ‘dormant’, so that it no longer makes any connection with the source frame [70], may be reawakened by AI-generated images prompted by verbal expressions of it. The role of synthetic images in constituting metaphorical meaning has particularly strong implications in the political arena where the metaphors in question concern marginalised and disempowered social groups. This paper investigates two metaphors that figure widely in anti-immigration discourses: immigration is flood versus immigration is invasion.

## 3. Pilot

To investigate the images that Gen-AI produces in response to flood versus invasion metaphors for migration, an initial pilot study was conducted for exploratory purposes. Qualitative observations emerging from the pilot data are then used to inform the design of the main study. The pilot study targeted a single model via Google Gemini. A zero-shot single prompting method was used requesting 15 images per metaphorical condition (30 in total) without providing any further information or feedback. The images were generated in a single sitting on 21 February 2026. The prompts used were:

### Create fifteen images depicting a migrant flood

#### Create fifteen images depicting a migrant invasion.

The images were returned as the montages displayed in Figs 1 and 2. Each montage included one duplicated image which is discounted from the analysis. A number of observations can be made of the two image sets. Both image sets appear to use a particular colour palette that is made up of browns, blues and greys. Although it is not clear that there is a discernible difference in the colours presented by each image set, colour is an important semiotic resource [71] and previous research has shown that dark blues, browns and greys emerge as dominant colours in AI-generated images of war [17] and post-apocalyptical video games [72].

**Fig 1:**
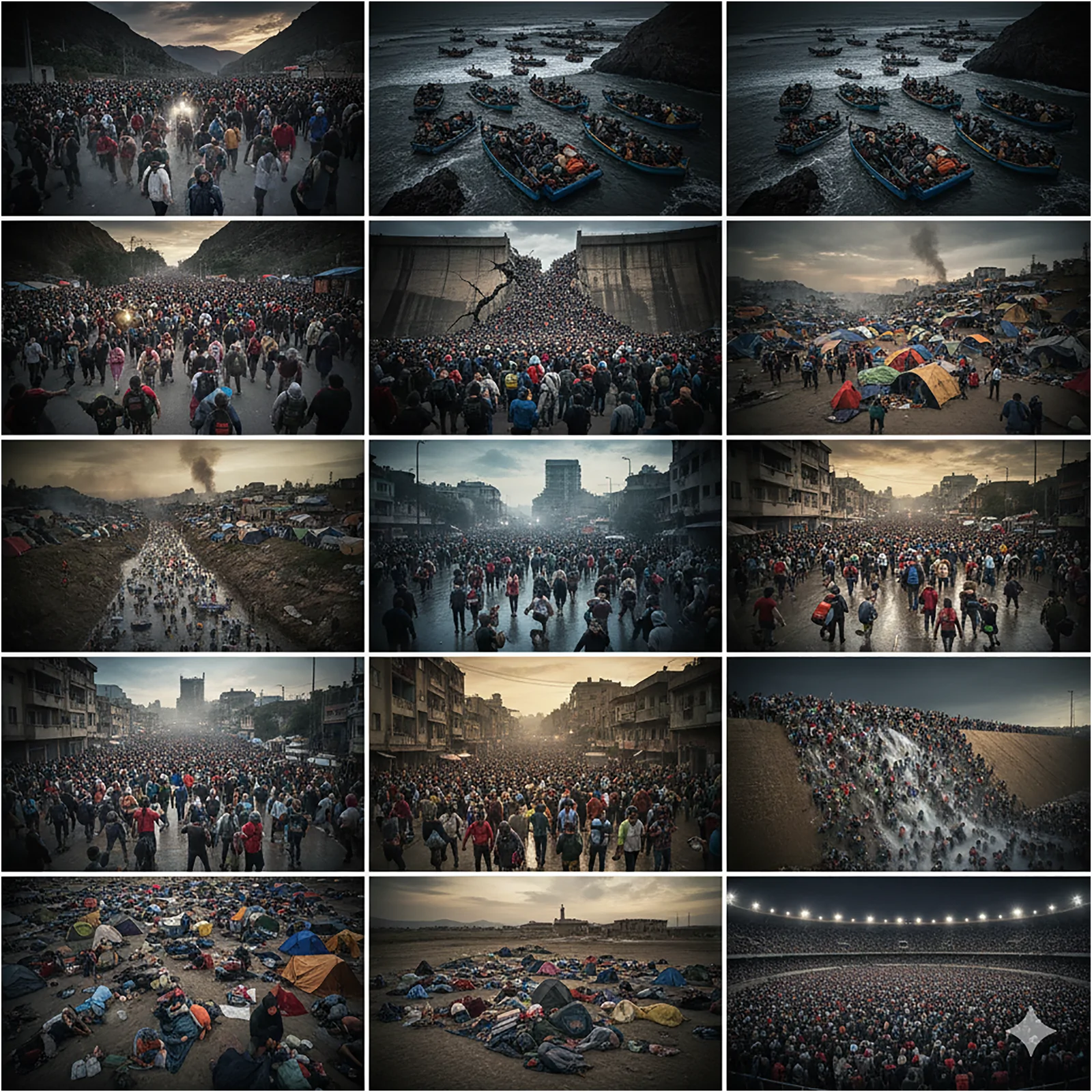
Images prompted by flood frame.

**Fig 2:**
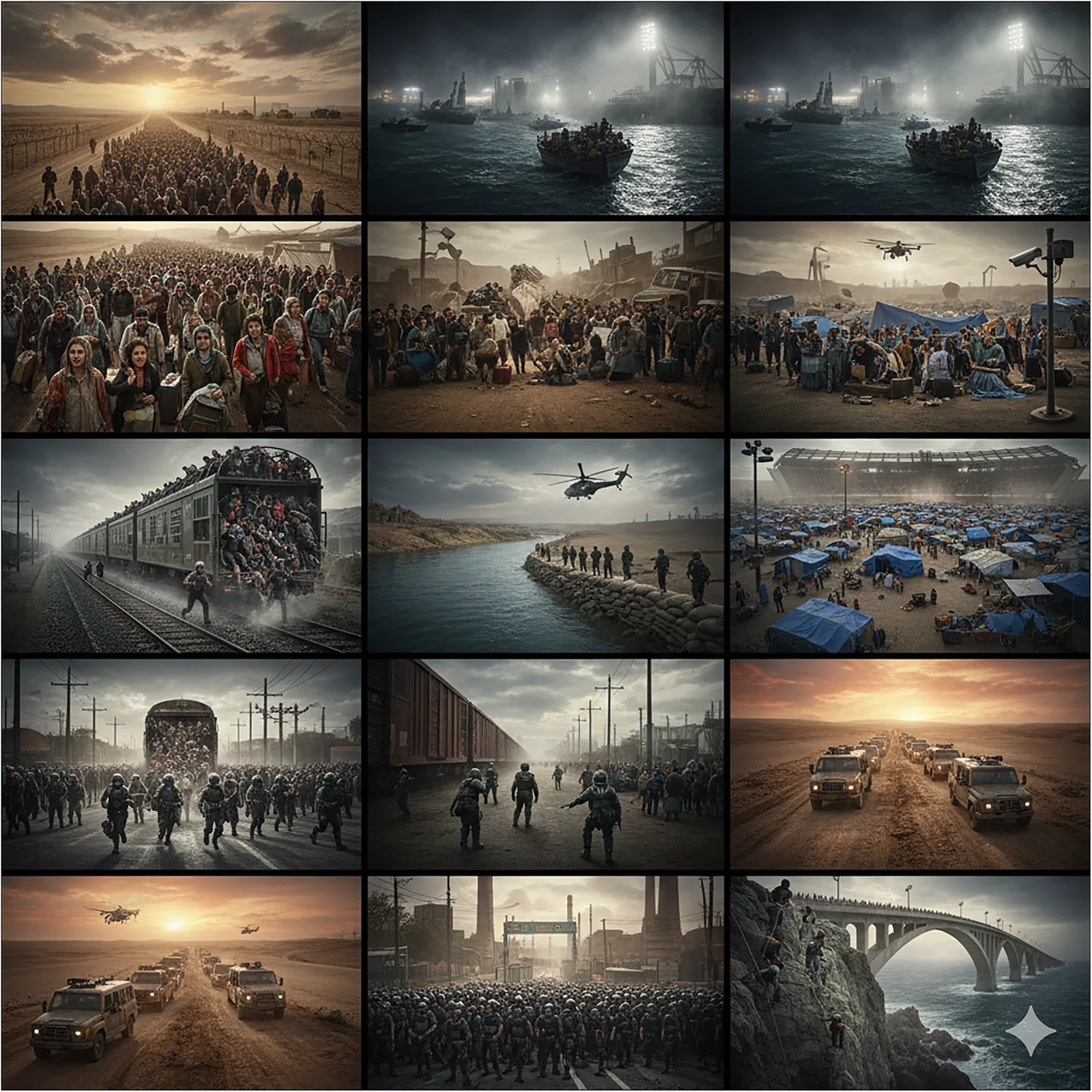
Images prompted by invasion frame.

Both data sets include images where migrants themselves are not shown or are not the main object of attention. For example, in three of the fourteen flood images (21.4%) and one of the fourteen invasion images (7.1%), migration is represented by temporary accommodation in the form of tents. When they are depicted, in nearly all cases migrants are shown in large quantities. The only exception is the image in the invasion set that shows a small number of people scrambling on cliffs by the sea.

Some notable contrasts between the two image sets can also be seen. For example, water is clearly visible in at least five of the flood images (35.7%) but in only three of the invasion images (21.4%). In the flood images, different relationships between migrants and water can be observed. For example, in two images, migrants are shown on board boats travelling by river and sea. In other images, streets are submerged in water which migrants are shown standing in or wading through. In the remaining image, migrants appear to be part of a waterfall cascading down a bank. This image can itself be read as figurative with the immigration is flood metaphor expressed through the *integration* of migrants and water. This contrasts with another image that can also be read as expressing the immigration is flood metaphor but which does not directly depict water. Instead, in this image the metaphor arises through the *substitution* of water with migrants who are shown ‘bursting’ through a dam-like structure (in Forceville’s [73] terms, the visual metaphors expressed by these images are examples of *hybrid* versus *contextual* metaphors respectively). In the invasion set, one image also shows migrants on board a boat in the sea. However, the boat is surrounded by other ships that are suggestive of a naval fleet. Indeed, the military are explicitly represented (through soldiers, vehicles, drones) in eight of the invasion images (57.1%) but none of the flood images. This contrast suggests that Nano Banana Pro is parsing the prompt compositionally to produce creative images that can either themselves be read as figurative or are otherwise consistent with the metaphorical prompt in referencing elements associated with the source-frame. A further difference concerns style with several of the images in the invasion set presenting in a way that is characteristic of film or videogame scenes.

## 4. Methods

### 4.1 Data

The main study targeted four models: GPT-Image-1.5, Flux 2 Pro, Gemini 2.5 Flash Image (“Nano Banana”) and Nano Banana Pro (Gemini 3 Pro Image). The four models were selected based on accessibility as well as ranked position defined by Elo scores, which capture end-user preferences (https://artificialanalysis.ai/image/leaderboard/text-to-image). All four models lean toward photorealism while other models like Midjourney lean more toward surrealism and other artistic styles. Models were accessed via a single platform Pixella.ai (https://pixella.ai/). Pixella.ai is a browser-based design platform offering a range of creative tools including TTI generation. (Pixella.ai offers an initial free trial period that leads automatically to a paid subscription unless cancelled. The free trial period includes a limited amount of credits that can be topped up for a one-off fee. Each image generation uses 10 credits.)

To generate the dataset, a zero-shot multiple prompting method was used. This means that for each model, the same prompt was posed multiple times without any guidance or feedback being provided between requests. Four simple prompts were created that crossed source-frame (flood vs. invasion) with NP structure (NN vs. N of N): migrant flood; flood of migrants; migrant invasion; invasion of migrants.

Prompts were kept intentionally simple to isolate the metaphorical expression and to minimise interference. No feedback was given between requests to avoid influencing the output in any way that might compromise validity (e.g., steering the model toward a particular image type that confirms expectations or meets aesthetic preferences).

It might be assumed that the same prompt would produce the same result on separate occasions. Early research into LLMs (as well as media coverage) has therefore been content to probe a tool just once and hold the response up as evidence of what Gen-AI does with a given prompt [34]. This, however, is not how Gen-AI works and, to identify any meaningful patterns in the images Gen-AI produces, the same request must be run multiple times. As Gillespie ([34]: 3) states, “social researchers investigating generative AI should be testing the same prompt over many instances, to understand not just whether these tools can generate three-dimensional or stereotypical representations – but how often”. Each prompt was therefore posed 5 times per model. This produced a final data set comprising 40 flood images and 40 invasion images.

### 4.2 Coding

To analyse the images a codebook was developed based on observations from the pilot study as well as prior research investigating visual representations of migration [49,74]. Any scene can be separated into *figure* versus *ground* components. The figure is the focal or topical object and the ground is the setting in which the figure occurs. Figure properties include its size, shape and orientation. In images of migration, features of the ground include landscape, walls and fences and the presence of other actors. An important property of the image as a whole is colour. Coding was performed automatically in the case of colour and manually for all other variables. Manual coding was performed by the author and independently by a second coder. The coding scheme is presented in Table 1.

**Table 1:** Coding scheme.

Colour	Hue	0-360°
	Saturation	Muted vs. vibrant vs. neutral
	Lightness	Dark vs. mid vs. light
	Contrast	High vs. low vs. normal
	Exposure	Bright vs. dark vs. neutral
Figure Properties	Size	Small (<20) vs. large (>20)
	Shape	Crowd vs. straight/curved/wavey line
	Orientation	Toward vs. away vs. across vs. mixed/unclear
Ground Elements	Environment	Urban vs. built vs. natural
	Fire	Present vs. not present
	Water	Present vs. not present
	Flags	Present vs. not present
	Walls / fences	Present vs. not present
	Military personnel / vehicles / equipment	Present vs. not present
	Multimedia	Present vs. not present

Forty per cent of the data (32 images) was independently coded by a second coder. Intercoder reliability was overall almost perfect with a mean kappa score of 0.873. Scores for individual variables ranged from substantial to perfect agreement. The kappa scores for individual variables were as follows: Quantity (1.000); Arrangement (0.913); Line Shape (0.828); Orientation (0.859); Environment (0.899); Flags (1.000); Security Walls/Fences (0.742); Military Personnel (0.796); Water (1.000); Fire (0.784); Multimedia (0.784). Since intercoder reliability was high for all variables, analyses proceeded on the original coding. All data and the project code book are available via the Open Science Framework at the following link: https://osf.io/zxq4c/overview?view_only=50a6cafc768a43029f86059e6f60d0b3.

### Colour

Colour is an important semiotic resource with different colours having culturally specific symbolic associations with emotions, moods, gender identities, political ideologies and religious ideas [71,75,76]. An intuitive way of capturing colour is through the three-dimensional Hue, Saturation, Lightness (HSL) model. Hue represents what we commonly take to be pure colour categories and is defined by angle values on a 0–360° colour wheel. For example, hues in the 0–30° range are experienced as red while hues falling within the range 210–270° are experienced as blue (see Table 2). Hues can also be grouped according to warmth with red, orange, yellow and pink considered warm colours and green, cyan, blue and purple considered cool colours. Saturation refers to the intensity or purity of the colour and is measured by percentage. Low saturation gives more muted colours while high saturation gives more vivid or vibrant colours. Colours are coded as muted (0–20% saturation), neutral (20–70% saturation) or vibrant (70–100% saturation). Lightness refers to how light or dark a colour is and is also measured by percentage with 0% lightness corresponding to black and 100% lightness corresponding to white. Colours are coded as dark (0–25% lightness), mid (25–75% lightness) or light (75–100% lightness). Not every colour that we experience is distinguished on the colour wheel. For example, what we experience as brown is in fact an orange or red hue with low saturation and lightness levels while grey is achromatic meaning it lacks both hue and saturation and is defined only in terms of lightness where it sits somewhere between black and white. Images obviously contain multiple colours. Here, it is the dominant colour that is the unit of analysis. An online tool is used to automatically detect the dominant colour within each image and extract its HSL values (https://www.imagetoolo.com/tools/image-palette-generator).

**Table 2:** Colour hue and angles on the colour wheel.

Angle	Hue
0-30	Red
30-60	Orange
60-90	Yellow
90-150	Green
150-210	Cyan
210-270	Blue
270-330	Purple
330-360	Pink

In addition to the dominant hue, the overall contrast and exposure displayed by each image was also automatically obtained using an online tool (https://pixample.com/histogram-analyzer/). Contrast concerns the degree of difference between hues and lightness in an image and is coded as high, low or normal. Exposure refers to the amount of light let in to an image determining how bright or dark the image is.

### Figure properties

The figure can be analysed in terms of its size, shape and orientation [49]. All three properties are semiotically significant [77,78]. Here, size refers to the quantity of people represented. A quantity is classed as *small* if it features fewer than twenty people and as *large* if it features twenty or more people. Shape refers to the profile assumed by the figure. In images of migration, prototypical shapes are *crowd* vs. *line* formations with lines further distinguished as *straight* (no bend), *curved* (one bend) or *wavy* (more than one bend). Orientation concerns the alignment of the figure relative to the viewer. Orientation is coded as *toward, away* or *across* (or *mixed/unclear*). The values for orientation are necessarily ideals which images do not always perfectly align with. Orientation is therefore coded according the value that images most closely approximate.

### Ground elements

The ground can be characterised in various ways. One is in terms of the environment. Environment is coded as *urban* where it represents a densely populated human settlement (i.e., town or city), *built* where it contains human-made structures (like roads, bridges, walls and buildings) and *natural* where it is a non-human-made landscape (e.g., desert, forest or mountain range). Images may depict more than one type of environment but are assigned a single code observing the following hierarchy: urban > built > natural. So, for example, an image showing a desert landscape surrounding a city is coded as *urban*. Certain elements may also feature in the ground that are consistent with flood versus invasion frames, including water versus fire or smoke. Images are therefore coded for presence or not of these elements. Other potential ground elements coded as present or not are: walls and fences; flags; military personnel, vehicles or equipment; and multimedia.

## 5. Results

### Colour

*Hue*: The dominant colour in flood versus invasion images differs in terms of hue (see Figs 3 and 4). The average hue for flood images is 31.3° while for invasion images it is 213.6°. A Watson-Williams test for circular means confirms that this difference is significant (F (1, 78) = 23.6, *p* < .001). A Mardia-Watson-Wheeler test also confirms that there is a significant difference in distribution with invasion images being more tightly clustered within the cyan-blue range and flood images being more spread across the red-yellow range (*W* = 31.8, *df* = 2, *p* < .001). Flood images are therefore strongly associated with warm hues while invasion images are strongly associated with cool hues.

**Fig 3:**
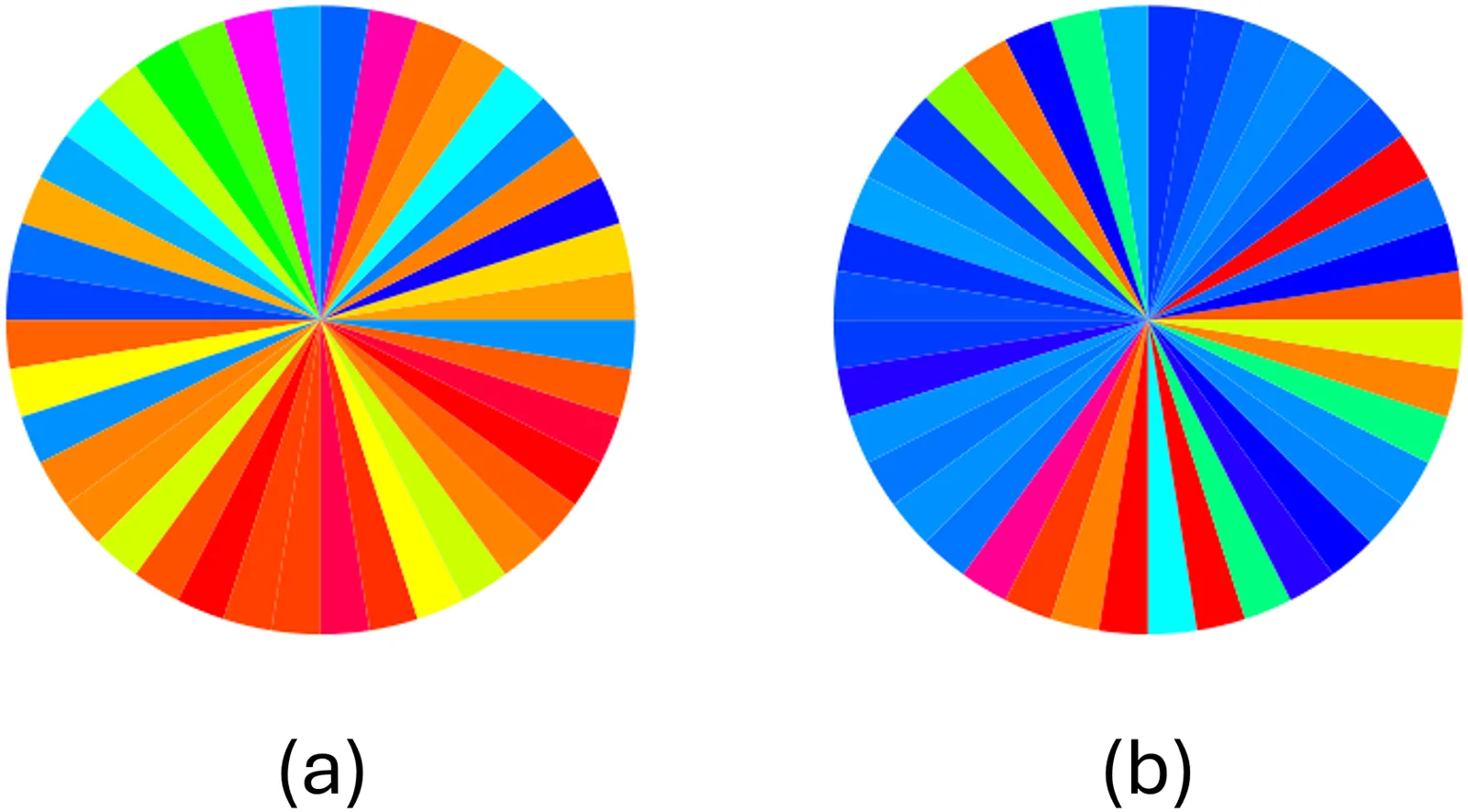
Differences in dominant hue of flood vs. invasion images. a = dominant hues of flood images; b = dominant hues of invasion images.

**Fig 4:**
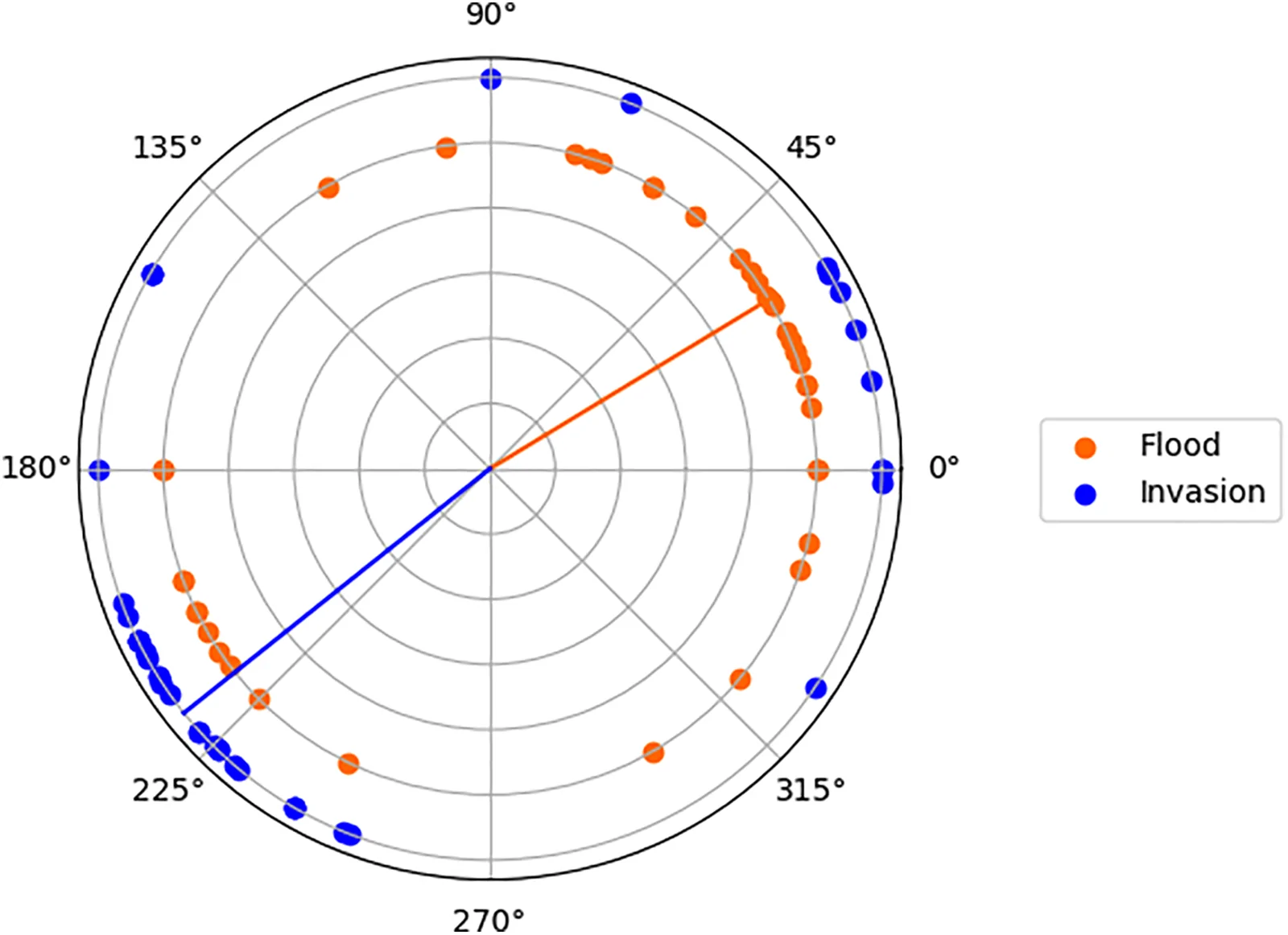
Distribution and circular means of dominant hue in flood vs. invasion images.

*Saturation*: In both image sets, the dominant hue displays low saturation and therefore appears as muted. The mean saturation level for flood images is 21.7% while for invasion images it is 22.6%. 62.5% of flood images and 57.5% of invasion images present a muted colour. In only 5% of flood and 5% of invasion images is the dominant colour vibrant.

*Lightness*: In both image sets, the dominant hue displays low lightness levels. The mean lightness level for flood images is 34.9% while for invasion images it is 35.1%. However, when treated as a nominal variable a significant difference emerges with invasion images more strongly associated with dark colours (67.5%) than flood images (50%) (χ^2^ = 6.665, *p* = < .05).

*Contrast and Exposure*: Both flood and invasion images display normal levels of contrast in the majority of instances (70% of images in each set). However, there are significant differences in exposure. While both sets are predominantly associated with normal levels of exposure, low exposure resulting in darker images is a more prominent feature of invasion images (27.5%) compared to flood images (10%) (χ^2^ = 4.021, *p* < .05).

### Figure properties

*Size:* There are no significant differences in figure size between metaphorical frames with migrants being represented almost exclusively in large quantities across both flood (97.5%) and invasion (87.5%) images (see Fig 5).

**Fig 5:**
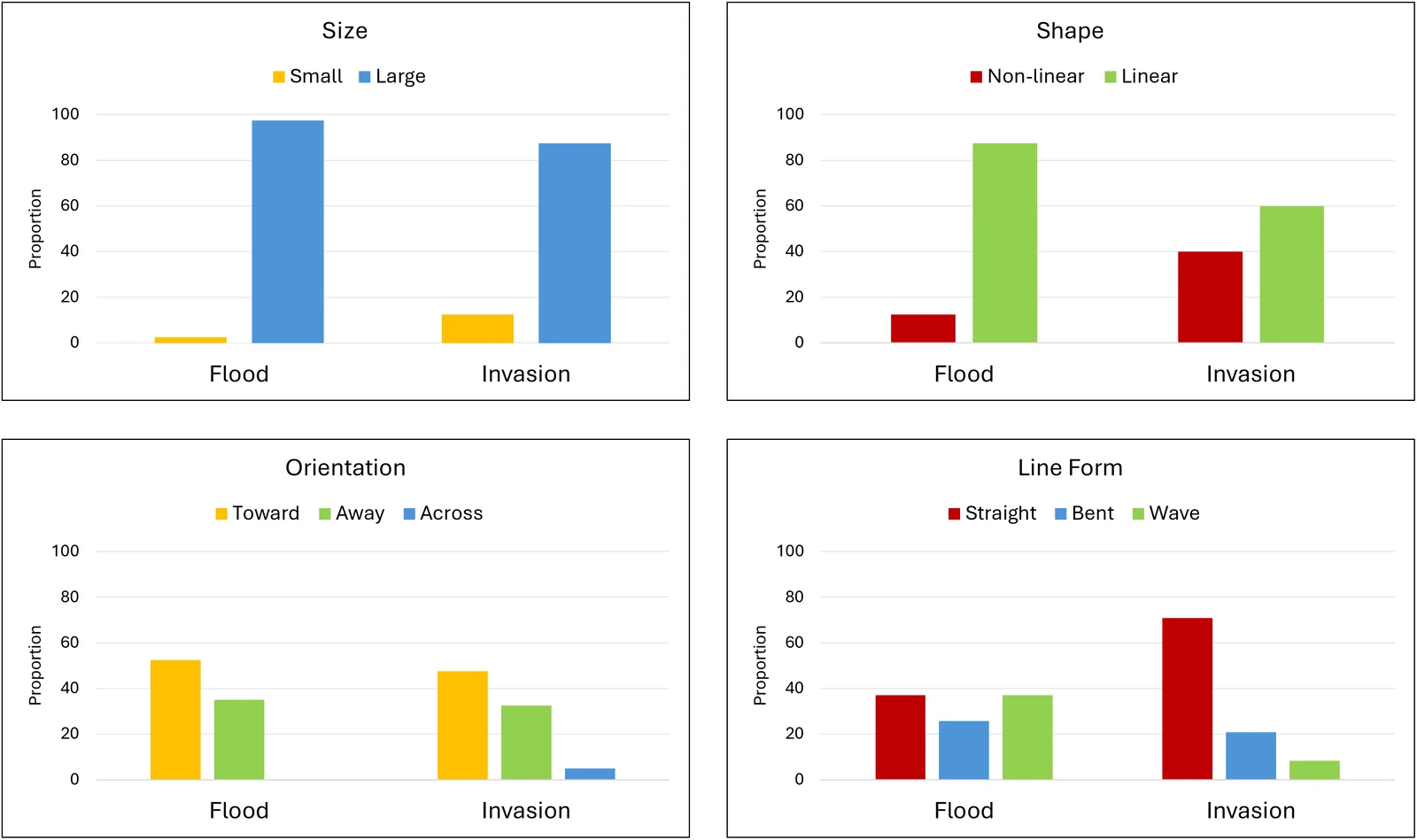
Figure properties by metaphorical prompt.

*Shape:* Migrants are arranged in a linear shape more frequently than a non-linear shape in both image sets but flood images are more strongly associated with linear forms (87.5%) than invasion images (60%) (χ^2^ = 7.813, *p* < .01) (see Fig 5). Where a linear shape is presented, flood images are more strongly associated with curved and wavey lines (62.9%) compared to invasion images (29.2%) while invasion images are more strongly associated with straight lines (χ^2^ = 15.505, *p* < .001).

*Orientation*: An orientation toward the viewer is the most frequent for both flood (52.5%) and invasion (47.5%) images with no meaningful difference between them (see Fig 5). However, when combined with shape, an image type emerges that is almost exclusively associated with the invasion framing (n = 5, 83.3% of all instances). In this particular image type, migrants are arranged in line formation, uniformly dispersed, and directly facing the viewer with the front of the line occupying the full frame-width at the bottom of the image.

### Ground elements

*Environment*: There is a significant difference in the type of environment depicted in images prompted by flood versus invasion metaphors. Most notably, urban environments feature more frequently in invasion images (65%) compared to flood images (27.5%), while natural environments feature more frequently in flood images (20%) compared to invasion images (5%) (χ^2^ = 12.136, *p* < .01) (see Fig 6).

**Fig 6:**
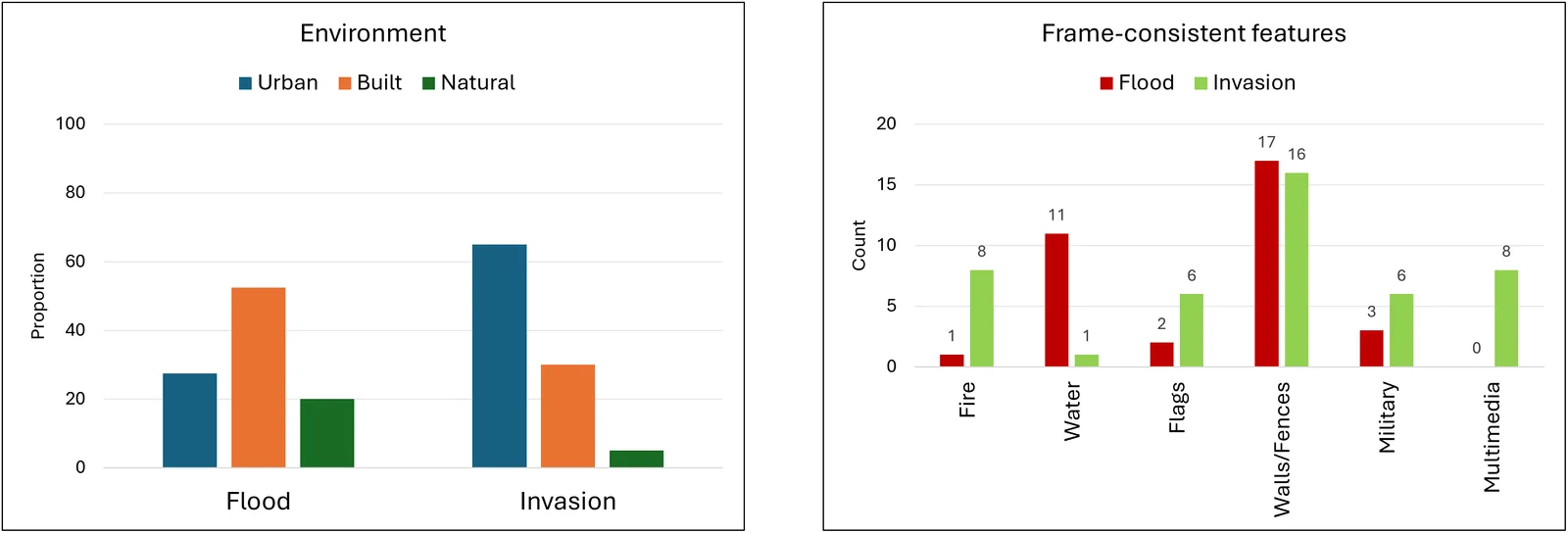
Ground elements by metaphorical prompt.

*Frame-consistent features*: The counts for frame-consistent features are relatively small (see Fig 6). However, a Fisher’s Exact test confirms reliable differences for fire (*p* < .05), water (*p* < .01) and multimedia (*p* < .01). Flags and the military occur more often in invasion images compared to flood images, although the counts are too small to show any statistical significance. Walls and fences feature equally saliently in both image sets.

### Variation by model and construction

Model had a significant effect on the images produced across a number of features as summarised in Table 3. Construction had a significant effect on only a single variable with walls and fences occurring in 60% of images prompted by the [NN] construction and in 22.5% of images prompted by the [N of N] construction (χ^2^ = 11.605, *p* < .001).

**Table 3:** Variation by model.

Variable	Sig.	*p* <	Comment
Colour			
Dominant hue	x		
Saturation	✓	.05	Flux 2 Pro produced fewer muted hues than other models and accounts for 100% of all instances of vibrant hue
Lightness	✓	.05	Nano Banana Pro produced more light hues than dark hues while all other models produced more dark hues than light hues
Contrast	✓	.001	Nano Banana Pro produced more high contrast images than normal or low contrast images while all other models produced more normal contrast images
Exposure	x		
Figure Properties			
Size	✓	.01	Flux 2 Pro produced more small group images than other models and accounts for 83.3% of all instances
Shape	✓	.01	Flux 2 Pro and GPT-Image-1.5 produced more non-linear formations than other models and account for 80.9% of all instances
Orientation	✓	.001	Nano Banana Pro produced more away images than toward while all other models produced more toward orientations than away
Ground Elements			
Environment	✓	.001	Gemini 2.5 Flash Image and GPT-Image-1.5 produced more urban and natural environments than built environments while Flux 2 Pro and Nano Banana Pro produced more built environments than urban environments. Gemini 2.5 Flash Image and GPT-Image-1.5 produced more natural environments than other models accounting for 90% of all instances
Fire	✓	.05	GPT-Image-1.5 produced more images depicting fire than all other models accounting for 66.6% of all instances
Water	✓	.05	GPT-Image-1.5 produced more images depicting water than all other models accounting for 58.3% of all instances
Flags	x		
Walls/Fences	x		
Military	x		
Multimedia	✓	.001	Flux 2 Pro produced more images depicting multimedia than all other models accounting for 100% of all instances

## 6. Discussion

### 6.1 General patterns

Migrants are represented almost exclusively in large quantities in images prompted by both flood and invasion metaphors. This is consistent with both framings which imply an excessive quantity or scale. In van Leeuwen’s [79] terms, migrants are *collectivised* rather than *individualised*. The contrast between the two forms of representation can be seen in Fig 7. Representations of migrants in large groups means reference “to their identity as individuals is avoided” ([80]: 10) and they are instead treated as an anonymised and homogenised mass, implying “that their life stories as individuals are not important” ([80]: 11). A sense of anonymity is further increased when the orientation of the figure is away from the viewer as in Fig 7(b) which discourages feelings of empathy or affiliation. Small vs. large group depiction is especially significant because migrants are judged as less capable of experiencing human emotions like tenderness, guilt and compassion when they are shown in large group sizes [81]. Large group depictions also lead to decreased perceptions of vulnerability [82]. Conversely, among people who are already high in threat sensitivity, anti-immigration attitudes are mitigated by images of individuals but not by images showing large groups of people [83].

**Fig 7:**
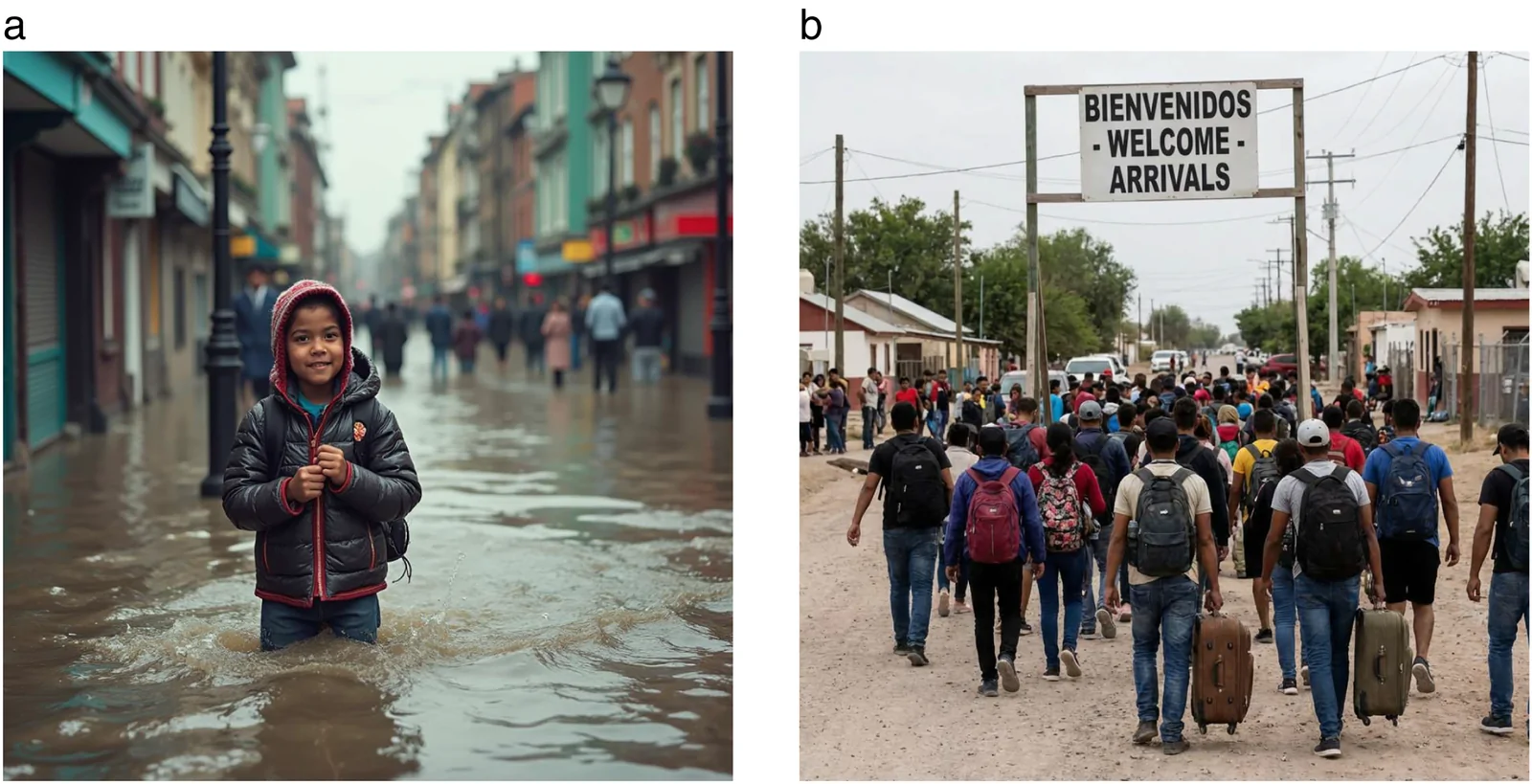
Individualisation (a) vs collectivisation (b).

In both sets of images, the predominant orientation of the figure is toward rather than away from the viewer, as in Fig 8. The effect is then not depersonalisation but a visual form of *proximisation* (cf. [84]) whereby the mass of people approaching the viewer creates a sense of impending threat.

**Fig 8:**
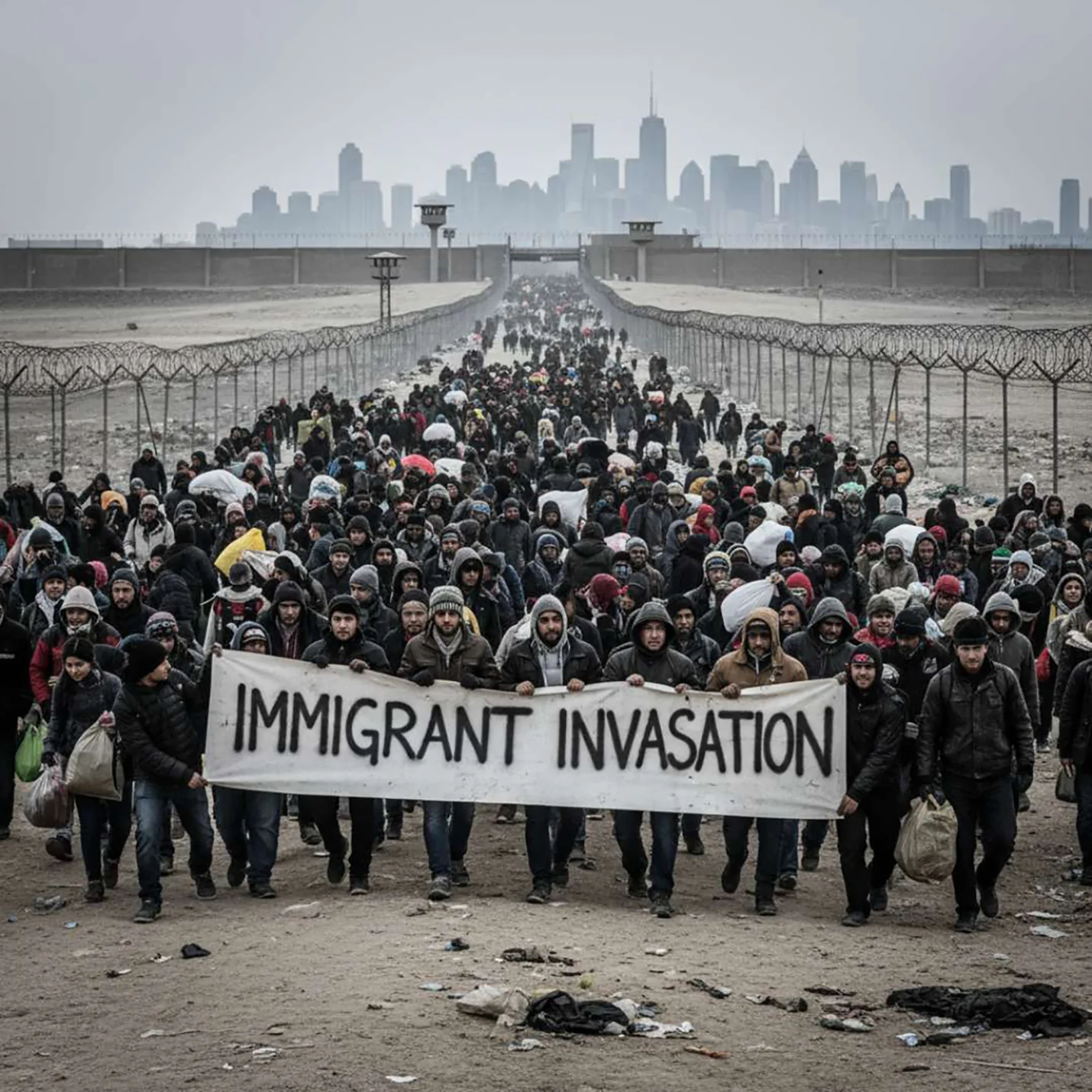
Orientation toward the viewer; low saturation and lightness.

The dominant hue in both flood and invasion images tends to display low lightness and saturation levels as in the example in Fig 8. The meaning-potential of colour dimensions like lightness and saturation relates to emotion [71,76,85]. The key affordance of saturation, for example, “lies in its ability to express emotive ‘temperatures’, kinds of affect” ([76]: 356). Dull and desaturated colour as in the image in Fig 8 creates an impression that is brooding or moody (ibid.). This is in contrast with images like the one in Fig 9 which have higher lightness and saturation values and connote more positivity or optimism.

**Fig 9:**
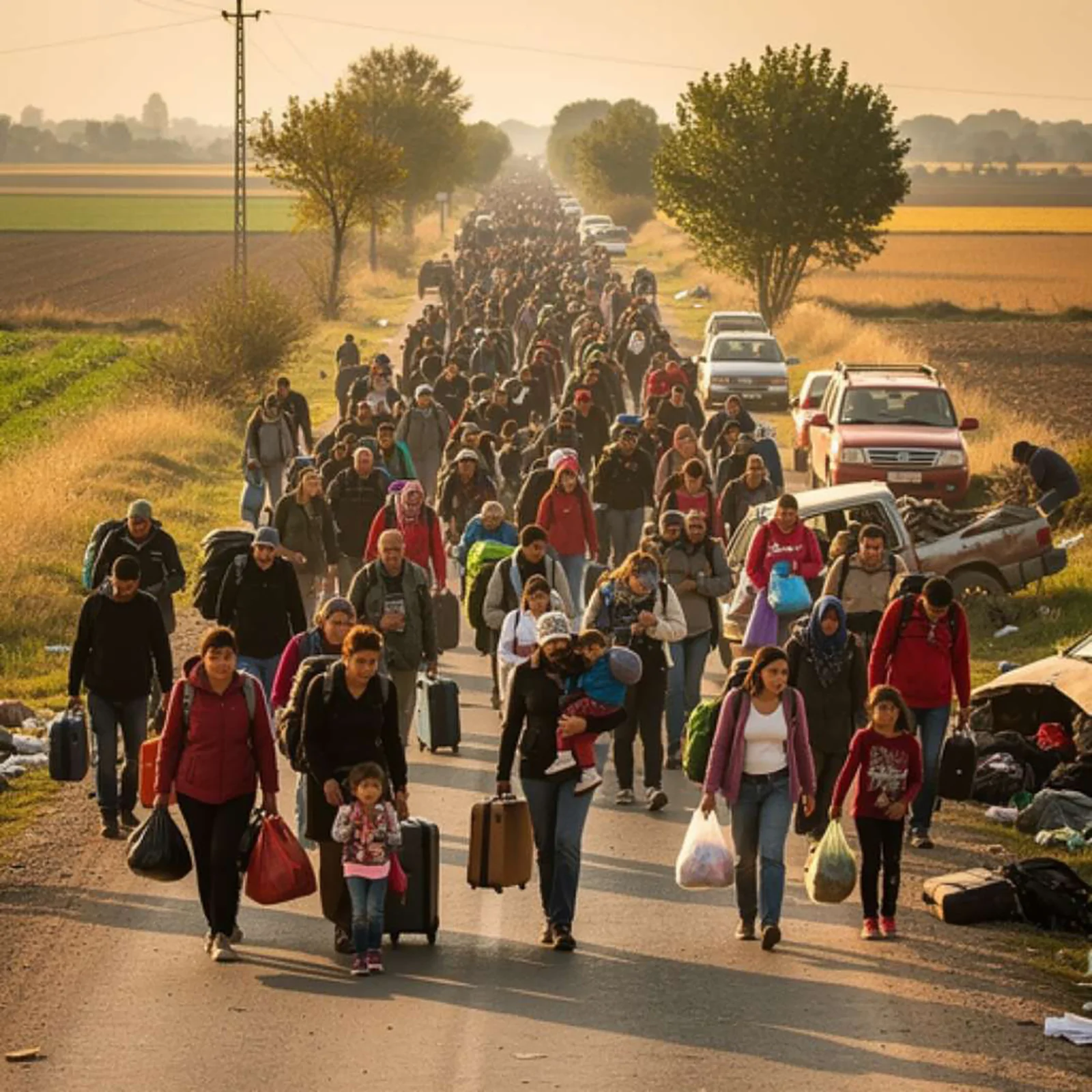
High saturation and lightness.

Walls and fences are a common visual trope in anti-immigration discourse [80,86] where they contribute to a *securitisation* of immigration [87–89]. Immigration is not inherently a security issue but becomes one when it is labelled that way by a securitising actor [90]. It is not only words but also images that provide securitising labels [91,92]. In immigration discourse, walls and fences function as securitising labels foregrounding security issues over other more humanitarian issues. As in the examples in Fig 10, walls and fences feature frequently in images prompted by flood (42.5%) and invasion (40%) metaphors, suggesting that both metaphors are part of a securitising discourse.

**Fig 10:**
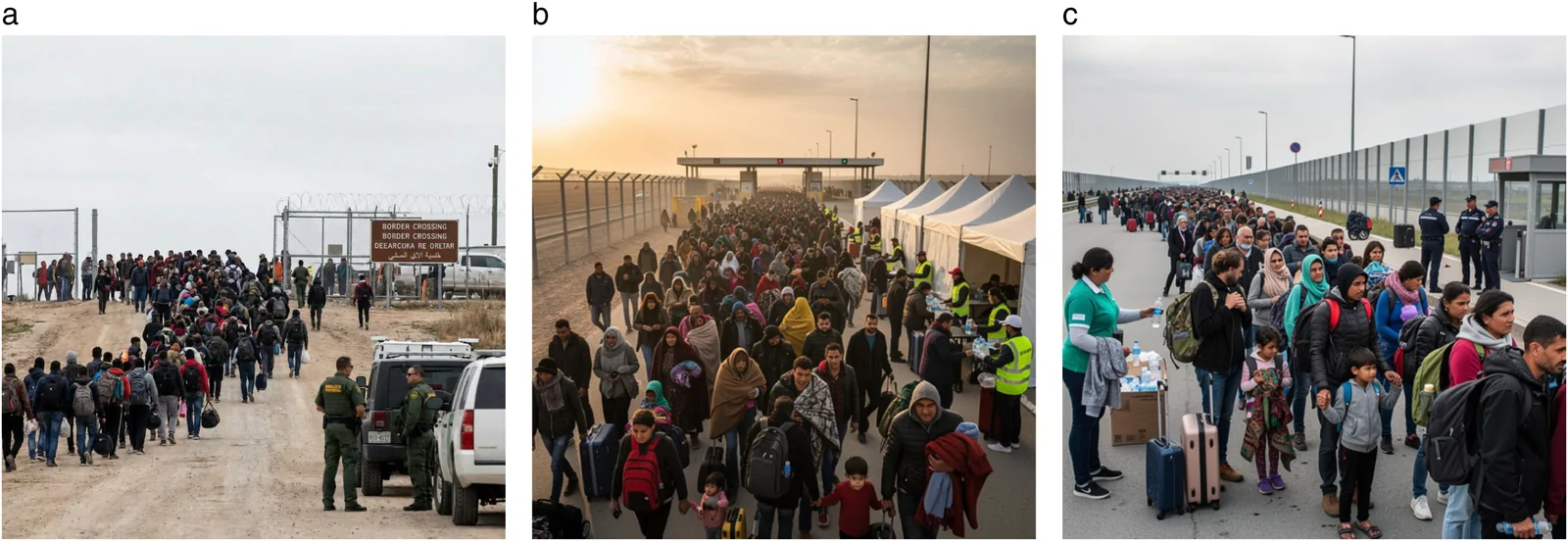
Securitising elements.

### 6.2 Flood images

Certain semiotic features emerge as characteristic specifically of images prompted by flood metaphors compared to images prompted by invasion metaphors. For example, images prompted by the immigration is flood metaphor are associated with red-yellow hues, natural environments and migrants arranged in curved or wavey linear forms. These three features combine to form ensembles like those in Fig 11 where the red-yellow hue of the dominant colour is accounted for by the desert-like landscape of the natural environment which the line of migrants is shown moving through.

**Fig 11:**
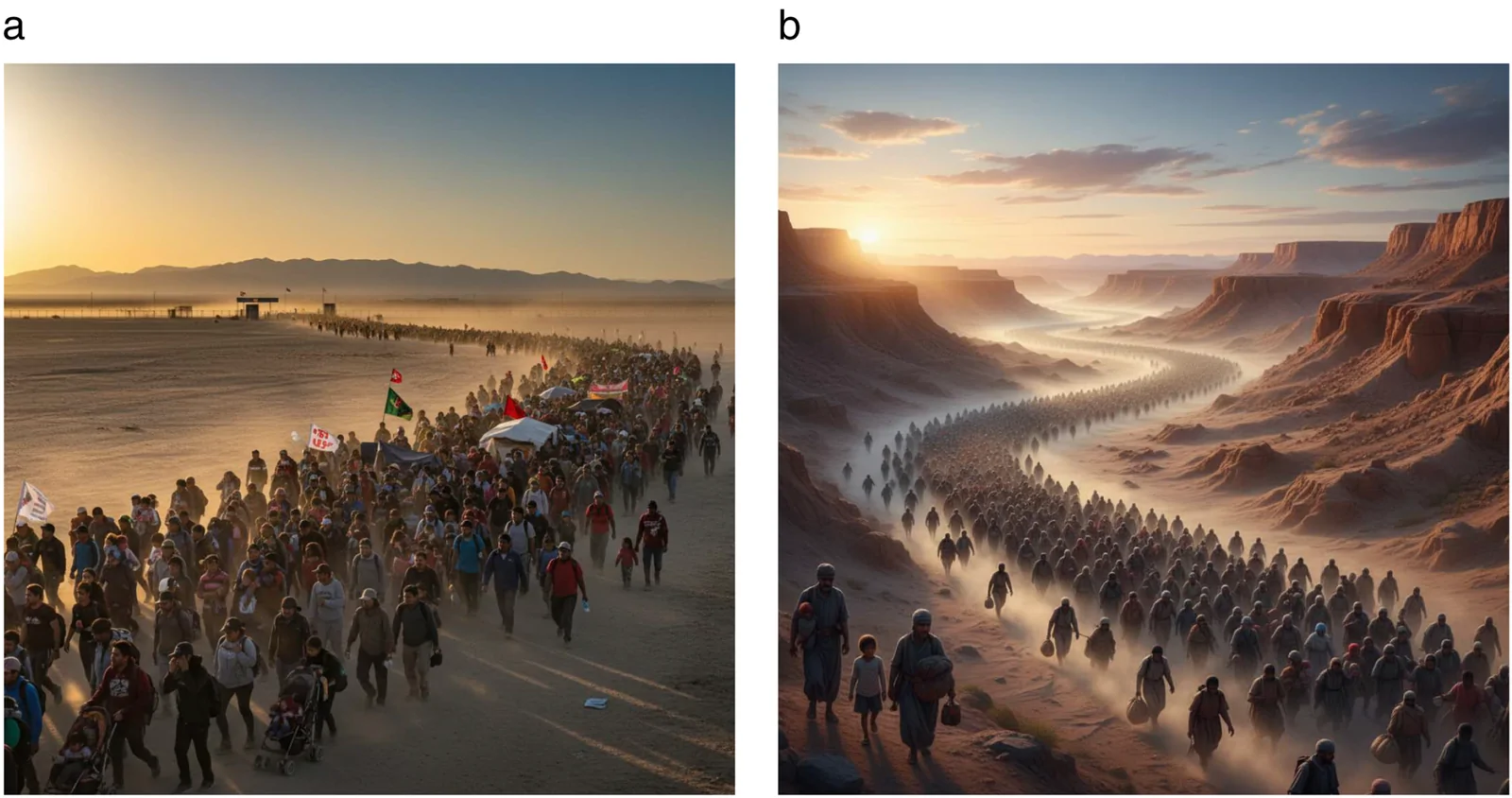
Line of migrants in natural environment. a = curved line; b = wavey line.

Images like those in Fig 11 may themselves be read as metaphorical where the overall ensemble bears a structural resemblance to prototypical images of rivers. This metaphoric potential is especially apparent, and thus likely to be realised, when the image is considered in conjunction with the text that prompted it. The images present examples of a type of visual metaphor that Forceville [93] calls *contextual.* In contextually rendered visual metaphors, an entity is turned into the target of a metaphor “by being depicted in a visual context in such a way that [it] is presented as if it were something else – the source” ([93]: 246). The metaphoric potential of images like those in Fig 11 arises where water is substituted with people who assume a form typically associated with waterways. When read metaphorically, the images produce a number of ideological effects. Construing people as a mass of moving water is naturalising, dehumanising and de-individualising. Moreover, the image of a river confirms a view that sees immigration as a continuous and indefinite problem. Verbally expressed water metaphors are shown to increase support for hostile immigration policies [53]. It seems likely that visual instantiations would achieve similar effects.

In images like those in Fig 11, water is not itself physically depicted but only implied by the shape of the figure and the context in which it occurs. However, images prompted by flood metaphors are also associated with literal depictions of water. Such images cannot be read metaphorically but are examples of frame-consistent images. In frame-consistent images, some element of the source-frame in a verbally articulated metaphor receives visual representation in the related image. A core element of the flood frame is obviously water. Images containing literal depictions of water are of predominately two types: showing people standing in or near still dirty water that is the residue of flooding, as in Fig 12a, or showing people struggling to move through a torrent of fast flowing water, as in Fig 12b.

**Fig 12:**
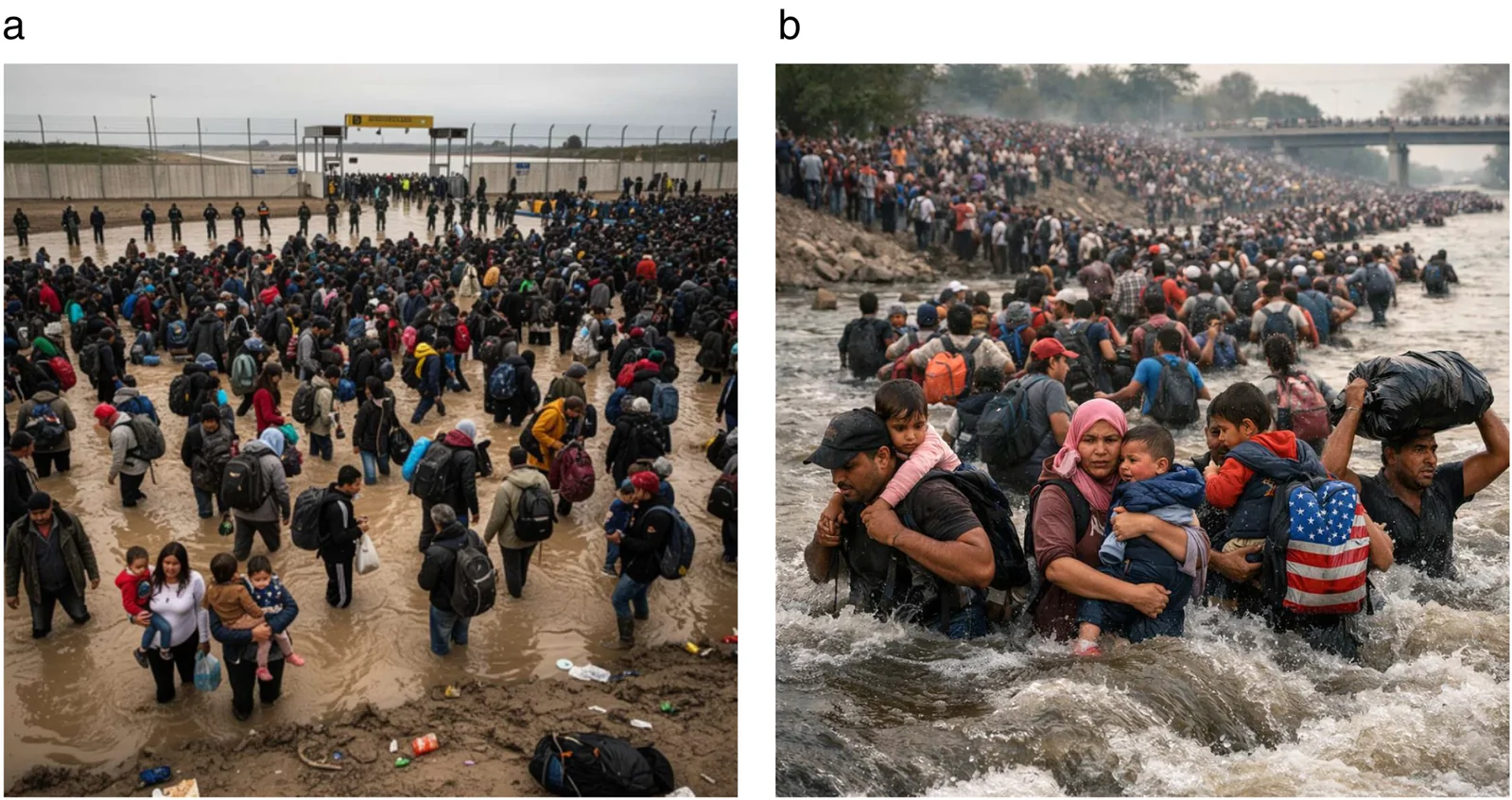
Frame-consistent images: Water.

For the images in Fig 11, the relationship between frame elements is *substitutive* with people replacing water where water would normally be expected. For the images in Fig 12, by contrast, the relationship is *additive* with people and water shown together. In both cases, however, it appears that TTI models are parsing metaphorical prompts compositionally to produce an image that draws from both source and target frames, combining them in different creative ways. It is worth noting that while images of the kind found in Figs 11 and 12a are produced by all four models, images of the kind found in Fig 12b are produced exclusively by GPT-Image-1.5 in response to the [N of N] construction.

### 6.3 Invasion images

A number of semiotic features also emerge as characteristic of images prompted by invasion metaphors which can be seen in contrast with those associated with flood metaphors. For example, while the dominant hue in flood images is typically in the red-yellow range, in invasion images it is typically in the cyan-blue range. Muted blues are shown to emerge as the dominant colour in other war-related media, including the post-apocalyptic video game *Fallout* where it is “frequently associated with military or nationalistic themes” [72]: 1). Likewise, while in flood images migrants are more frequently arranged in curved or wavey line formations reminiscent of rivers, in invasion images they are more frequently arranged in straight line formations that resemble military marches or parades. And while flood images are more strongly associated with built or natural environments than invasion images, invasion images are more strongly associated with urban environments. Combinations of some or all of these features occur, together with a figure orientation toward the viewer and a specific form of stylisation, in a particular sub-set of images that recall post-apocalyptic movies or video games. In this image-type, exemplified in Fig 13, migrants are shown in a straight line formation, arranged with uniform dispersion, moving toward the viewer and typically away from a cityscape.

**Fig 13:**
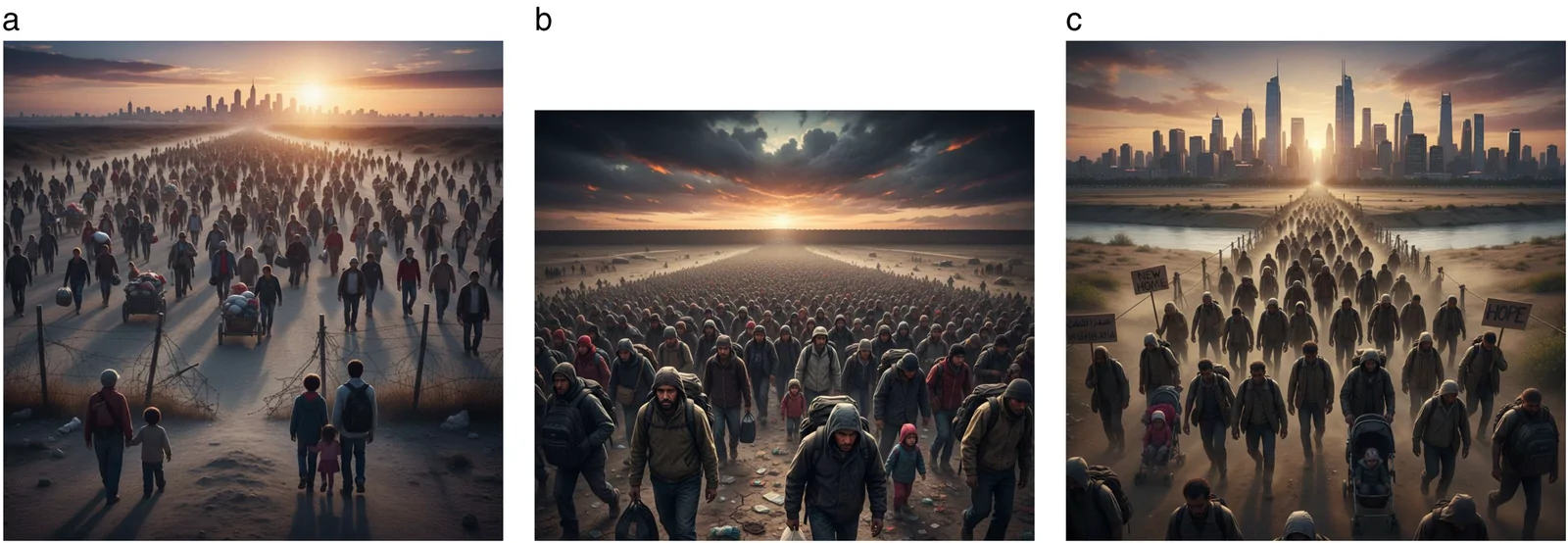
Post-apocalyptic images.

Intertextuality and interdiscursivity are important vehicles for visual metaphor. As Werner [94] states, “the echoing of themes, quotations, symbols, storylines, or compositional elements from older images and famous written texts may create visual metaphors”. Images like those in Fig 13 are capable of being read metaphorically as a function of the interdiscursive connections they make with post-apocalyptic movies and video games. Such interdiscursive connections are forged through compositional and stylistic features which the images have in common with scenes associated with these alternative genres. As a consequence of the mapping invited, immigration in the real world is likened to post-apocalyptic scenarios in movie or videogame ‘worlds’ where entire populations are displaced. Of course, the metaphorical interpretation here is dependent on the reader’s familiarity with the genres alluded to.

A notable feature of these images is perspective where the figure is orientated directly toward the viewer. Kress and van Leeuwen [77] argue that the difference between detachment and involvement with respect to represented actors in an image is a consequence of angle where “the frontal angle is the angle of maximum involvement” (p.145). While an oblique angle implies that depicted actors are not part of ‘our’ world, the frontal angle implies that they are part of ‘our’ world or soon will be. The low exposure found in these images further contributes a sense of foreboding. Of the six images that fall into this specific sub-category, it is worth noting that the majority (83.3%) are produced by Gemini 2.5 Flash Image.

Other features that show up specifically in images prompted by invasion metaphors are fire and multimedia. As water is a core element of the flood frame, so fire is a salient aspect of the invasion frame. Images like the one in Fig 14 are less likely to be read metaphorically but are consistent with the metaphorical framing in the prompt where a salient source-frame element receives visual representation.

**Fig 14:**
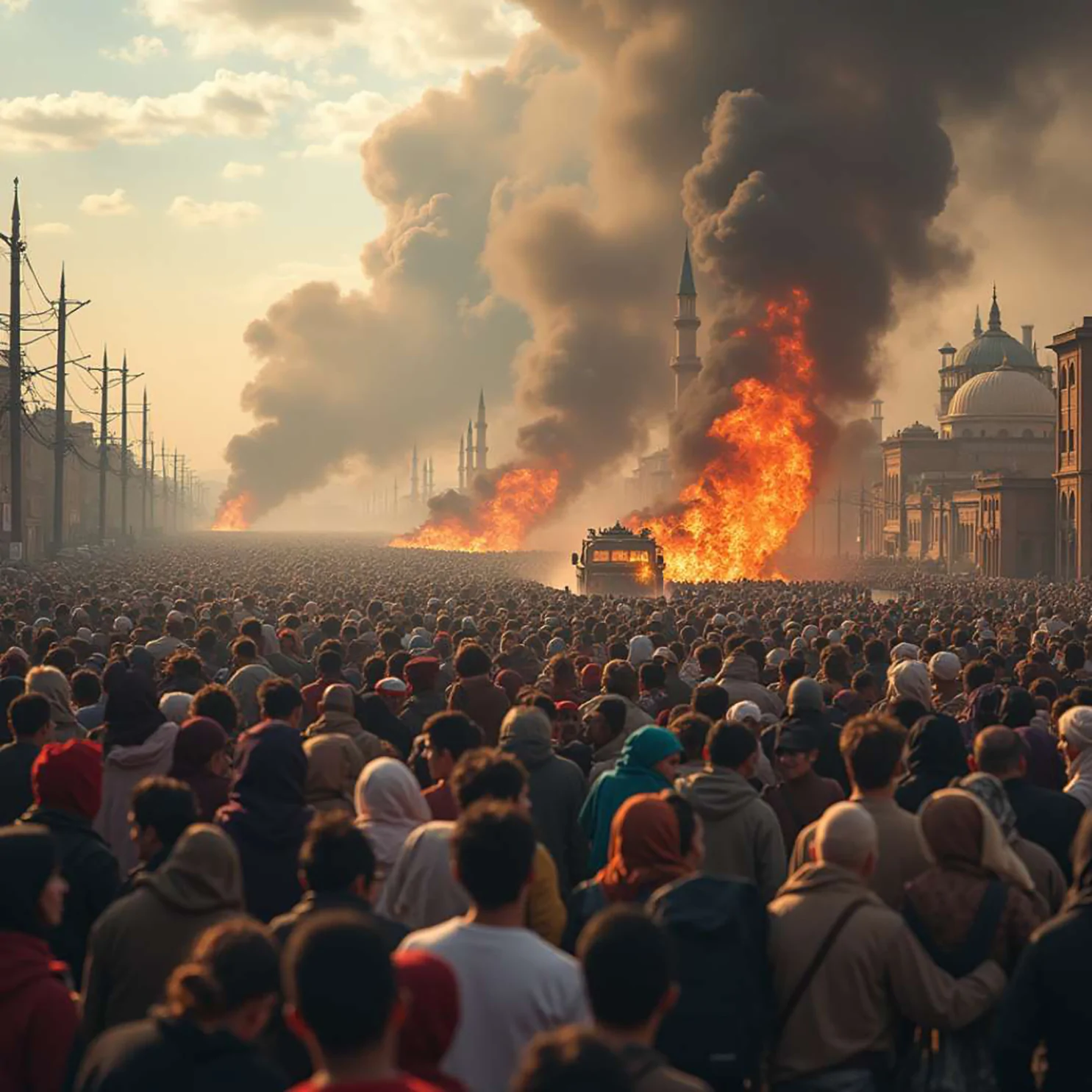
Frame-consistent image: Fire.

Multimedia is not an element specific to the invasion frame but is present in 20% of invasion images and does not occur at all in flood images. Examples are given in Fig 15. In Fig 15a, a multimedia screen is displayed within the image. In Fig 15b, the image is itself a multimedia screen as indexed by the news ticker which is a feature of broadcast television news. Indeed, six of the eight images that include multimedia are related to news communication showing either digital billboards displaying news bulletins or screens within TV news studio environments. The presence of multimedia in these images thus constructs or confirms for users the *newsworthiness* [95,96] of immigration. News values include impact and timeliness. The presence of news screens suggests ‘breaking news’, which serves to construct immigration as a currently unfolding crisis. Images including multimedia are produced exclusively by Flux 2 Pro.

**Fig 15:**
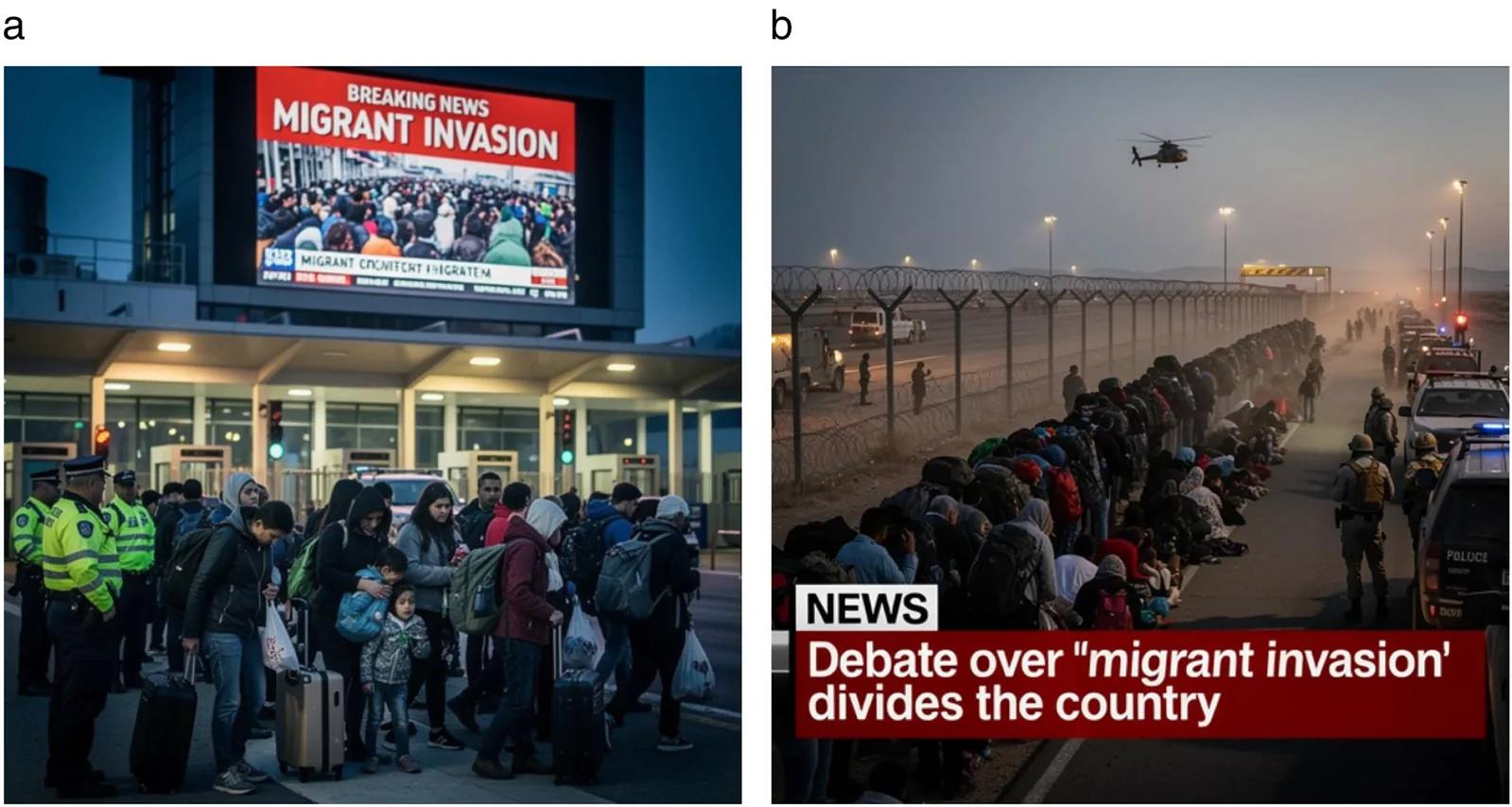
Multimedia in invasion images.

Other elements of the invasion frame include flags and the military. Although the counts are too small to be statistically reliable, flags occur three times as frequently in images prompted by the invasion framing compared to images prompted by the flood framing. Flags are also previously shown to feature in AI-generated images of war [17]. Flags are symbols of nationhood [97]. They can signify national identity but they can also be used to lay claim to territory or assert cultural dominance. The flags in the AI-generated images, exemplified in Fig 16, are notable for not being the flag of any particular nation but for all being red, a colour strongly associated with the flags of several modern Muslim-majority countries. The red flags may therefore symbolise a perceived cultural or corporeal threat coming from Muslim migrants in particular.

**Fig 16:**
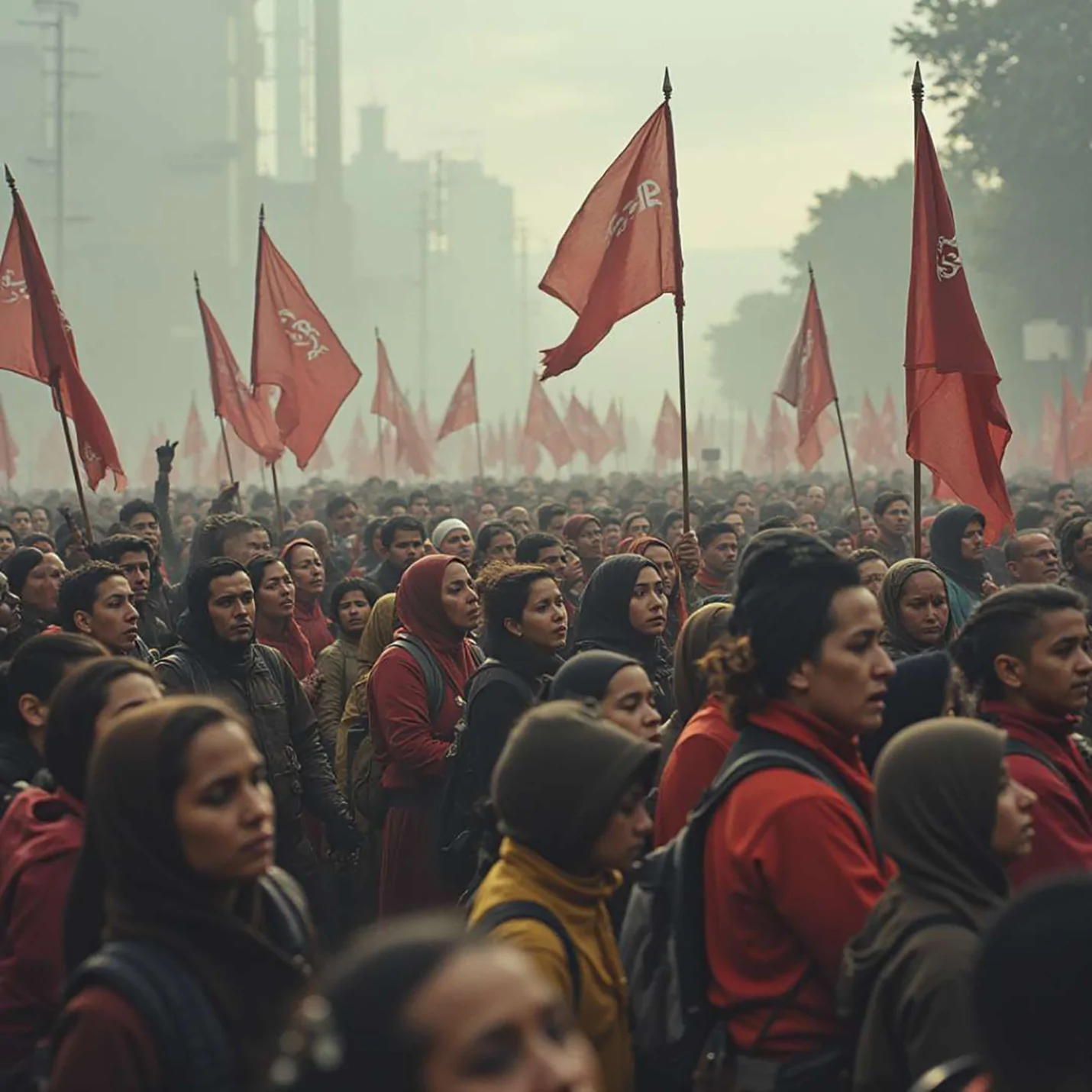
Flags.

Elements associated with the military, including military personnel, vehicles or equipment, occur twice as frequently in invasion images compared to flood images. Images featuring the military, as in Fig 17, do not instantiate an immigration is invasion metaphor but, rather, represent the conclusion of an argument based on this metaphor. As El Refaie ([4]: 368) states, “the logical consequence of regarding refugees as an invading army is to defend oneself and fight back” and this, within the constraints of the metaphor, means deploying the military. The images thus confirm a worldview, born of metaphorical ways of thinking, in which certain courses of action are seen as legitimate.

**Fig 17:**
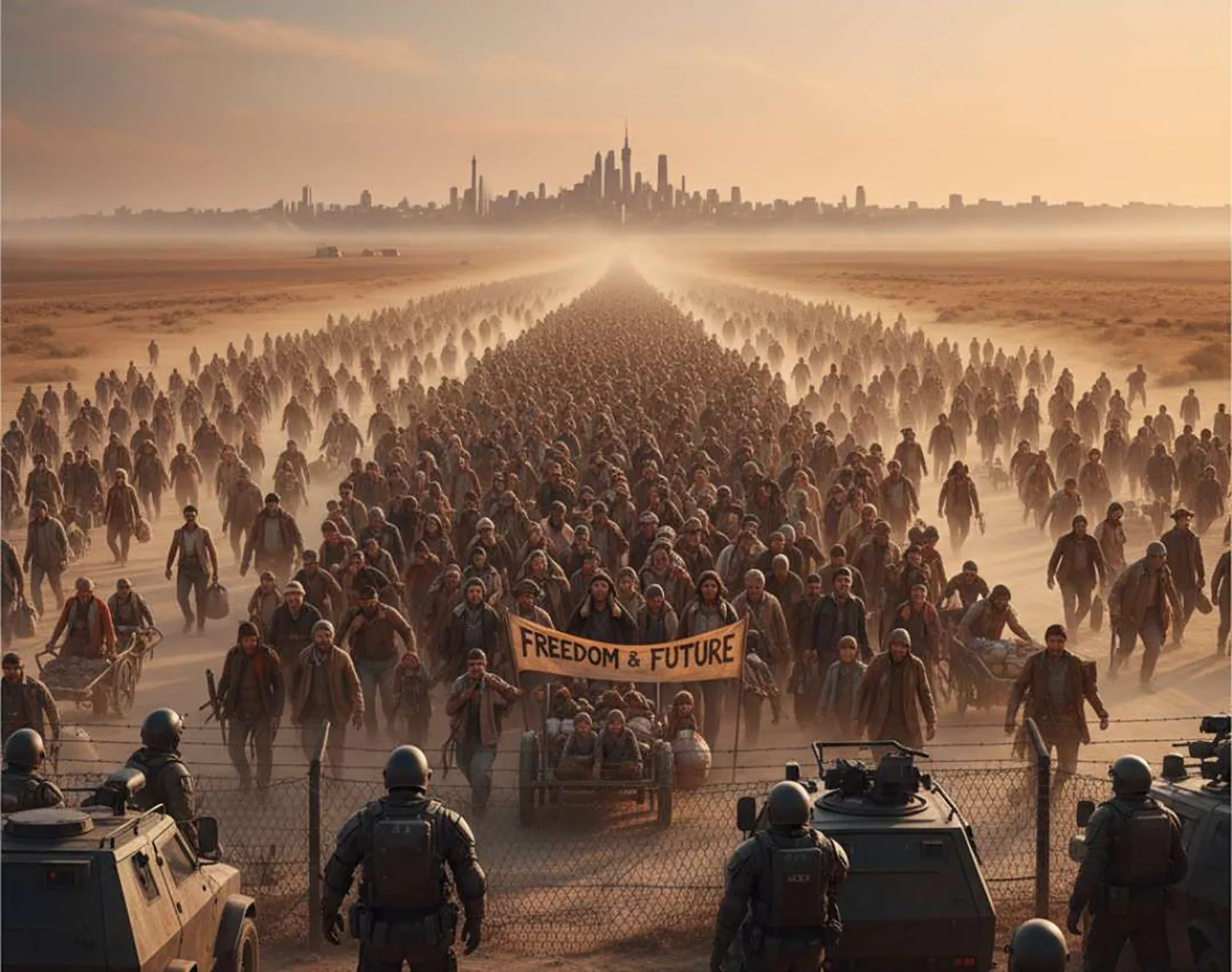
Military.

## 7. Conclusion

The discursive construction of social and political issues like immigration is increasingly mediated by AI-generated images – both as users encounter AI-generated images produced by others and as they engage TTI technologies to produce images for themselves. This paper has investigated the images that AI generates in response to flood versus invasion metaphors for migration.

Metaphor is a cognitive process of frame-projection in which a source-frame is mobilised to provide a template for sense-making with respect to a target-frame. Crucially, metaphor involves the construction of mental imagery. Increasingly, that mental imagery may be influenced by AI-generated images, especially among specific communities. For such communities, the meanings of a metaphor may come, in part, to be shaped by the synthetic images to which they are exposed.

Results show that AI produces a mix of realistic images reflective of the visual content that co-occurs with the metaphorical expressions and more creative images that combine aspects of both source and target frames in novel ways. This suggests that AI models parse metaphorical prompts both compositionally and non-compositionally. Where metaphorical prompts are parsed compositionally, images vary by source-frame with flood versus invasion metaphors prompting images that differ in light and colour, spatial arrangement, setting, and style, as well as inclusion of frame-consistent features like fire and water.

The images generated by AI in response to flood versus invasion metaphors may in some cases be read as visual articulations of the metaphor given verbally in the prompt and in other cases more minimally as frame-consistent images. When confronted with images generated by metaphorical prompts, users thus have confirmed for them the logics, perspectives and prejudices expressed in the metaphor. For users already immersed in far-right discourses, this is likely to have further interpellating effects and legitimise hostile attitudes and policy preferences.

The paper makes a contribution to the ongoing dialogue between critical discourse studies and cognitive metaphor research by providing empirical insights into the type of images generated by metaphorical expressions that feature saliently in anti-immigration discourses. However, several limitations can be identified which the study invites further consideration of in future research. For example, the study focussed on four models, which were found to behave in different ways. This shows that the effects of AI-generated images on metaphor understanding will vary across individuals depending on the models they engage with or are exposed to. Future research might include more models and more systematically investigate the differences between them. Likewise, the study collected data at a single point in time. However, the effects of AI-generated images on metaphor are likely to be cumulative and the algorithms on which TTI technologies rely are constantly evolving. Future research might therefore assume a longer temporal horizon and track model behaviour diachronically. Finally, of course, there are other discourses and other metaphors where the complex interaction between politics, ideology, multimodal semiotics and TTI technology may be investigated. It is hoped that the present paper can be built upon in a broader programme of research addressing changes to politics, culture and society brought about by the advent of Gen-AI.
